# Tinnitus: Characteristics, Need for Therapy, and Therapeutic Outcomes; Results of an International Patient Initiated Platform

**DOI:** 10.3389/fneur.2021.778450

**Published:** 2022-01-20

**Authors:** Adriana L. Smit, Markku Vesala, Hazel Goedhart, Job van Eijden, Christiaan Wempe, Inge Stegeman

**Affiliations:** ^1^Department of Otorhinolaryngology, Head and Neck Surgery, University Medical Centre Utrecht, Utrecht, Netherlands; ^2^University Medical Center Utrecht Brain Center, University Medical Center Utrecht, Utrecht, Netherlands; ^3^Tinnitus Hub Ltd, Amsterdam, Netherlands; ^4^Tinnitus Hub Ltd, London, United Kingdom; ^5^Department of Ophthalmology, University Medical Center Utrecht, Utrecht, Netherlands; ^6^Epidemiology and Data Science, Amsterdam University Medical Centers, University of Amsterdam, Amsterdam, Netherlands

**Keywords:** tinnitus, therapy, treatment outcome, survey, patients

## Abstract

**Introduction:**

So far, there is a gap of knowledge about factors influencing the impact of tinnitus, the need for treatment, as well as the experienced effect of regular and alternative tinnitus therapies. In this study, we analyzed the need for treatment and the outcomes of these treatments in an international patient initiated in tinnitus platform.

**Materials and Methods:**

Two surveys were undertaken at an online tinnitus patient support community (www.tinnitustalk.com). The surveys were aimed at (1) using tinnitus treatment and outcomes and (2) the factors that influence tinnitus. Univariable logistic and linear regression were used to calculate the relation between the factors and the tinnitus impact as well as the relation with the used tinnitus treatments and the outcomes.

**Results:**

Of the participants to the first survey (*n* = 5,017), 2,914 (58.1%) used one or more tinnitus therapies, whereas others most commonly self-administered sound therapy [*n* = 1,562 (31.1%)] and supplements/herbal medicines [*n* = 1,157 (23.1 %)]. Being female [odds ratio (OR) 0.83 (95% CI 0.74–0.93, *p* < 0.01)], tinnitus impact, and some degrees of hearing loss and hyperacusis were all statistically significantly associated with higher odds of having tinnitus treatment. Out of the second survey (*n* = 6,115), it was found that patient physical and psychological factors were statistically significantly related to tinnitus impact.

**Conclusion:**

In this study, we demonstrated the usage and experience of (multiple) tinnitus therapy in patients. Several patient physical and psychological characteristics were found to be related to tinnitus impact and therapy usage. These outcomes might function as the next step to find a personalized treatment and to improve the tinnitus health care.

## Introduction

Tinnitus is the perception of sound without an external stimulus, often experienced as a ringing or buzzing sound (1, 2). It is a common condition with an approximate prevalence of 5.1–42.7%, depending on the selected population and used definitions (3). While the underlying etiology of tinnitus is still debated, one hypothesis is that the tinnitus arises from changes in neural activity caused by reduced or lack of auditory input due to hearing loss which often accompanies tinnitus (4, 5). It is considered a complex condition, in which many components are responsible for perceived impact, such as loudness and tinnitus-related difficulties such as sleep and concentration problems. The impact people experience is diverse and the individual needs of patients for tinnitus-related health care varies.

So far, several tinnitus treatment modalities are available. Due to the lack of curative treatments, these therapies all focus on relief from symptoms as the highest achievable goal. Of these, psychological therapy such as cognitive and/or behavioral therapy (CBT) (4, 6–8) or the application of hearing aids in case of accompanying hearing loss (4) is a part of the standard clinical care in many countries to improve the quality of life or reduce tinnitus-related distress (9–11). Considering the fact that there is no evidence for the effectiveness of drug treatments specifically for tinnitus (11), it is of interest that an estimation of 4 million off-label prescriptions each year for tinnitus relief have been reported in Western Europe and the USA (3).

Due to the ongoing burden that people with chronic tinnitus are experiencing, some patients keep searching for a solution within regular and alternative therapies to solve their problem (12, 13). Thereby, there is a gap of knowledge about factors influencing peoples' tinnitus, the need for treatment, and the experienced effect of conventional and non-conventional tinnitus therapies. This is of special interest as people with tinnitus do not necessarily attend a physician in their search for tinnitus relief.

In order to improve the knowledge about a broad sample of individuals experiencing tinnitus, those with and those without burden, we assessed a worldwide internet-based cohort. We aim to analyse the factors of influence on the experienced tinnitus, the need for treatment, and the experienced effect of tinnitus therapy.

## Materials and Methods

### Ethical Considerations

Ethical approval for this study and a waiver of informed consent were obtained from the local ethical committee of the University Medical Center Utrecht, Netherlands (local number: 19-134/C), for anonymous data collection and with the intention to use these data for scientific analyses. This study was performed in accordance with the Declaration of Helsinki (version 2013, Fortaleza) and the Medical Research Involving Human Subjects Acts (WMO).

### Study Design and Setting

This study was conducted following a cross-sectional cohort design. By partnering with Tinnitus Hub to collect survey responses from members of their online patient support community, Tinnitus Talk (www.tinnitustalk.com), data of people experiencing tinnitus were retrieved. This platform was initiated in 2011 for worldwide peer-to-peer support services and is available for everyone without any costs and is written in English. Included in this study were two separate surveys conducted in February 2016 (Survey “Causes and Treatments”) and October 2017 (Survey “Physical Links”) on this platform.

### Study Aim

The primary aim of this study was to analyse the characteristics of those individuals with tinnitus who underwent treatment and compare these characteristics to those who did not undergo treatment. Secondly, we aimed to evaluate the influence of different variables on experienced tinnitus.

### Participants

Visitors of all ages of the website of the Tinnitus Talk platform were asked to fill out a questionnaire about their tinnitus and effect of treatments. Questionnaires were handled anonymously.

### Outcome Measures: Survey Causes and Treatments

The online questionnaire of the survey “Causes and Treatments” was aimed at the factors that influence tinnitus and the experience with tinnitus treatment. It contained 32 items and included questions about demographics, tinnitus characteristics, cause of the tinnitus, comorbidities, tinnitus-related conditions, the impact of tinnitus on daily life, physiological/physical factors of influence on experienced tinnitus, received tinnitus treatment, and outcomes. Questions were checkboxes in which the participants had to select the answer most applicable to their situation (Attachment 1 Survey “Causes and Treatments”).

Demographic variables included gender and age range. Tinnitus characteristics were scored with regard to tinnitus time of onset, pulsatile (described as tinnitus that is rhythmic), and somatic character (defined as that the tinnitus changes in volume by physical movement or touch). Comorbid conditions were asked for including hyperacusis (described as “a sensitivity to sounds, often you will think sounds are irritating and painfully loud when others hear them as normal”) and hearing loss (categorized in “none known of,” “mild hearing loss; may struggle a little to keep up with conversation,” “moderate hearing loss; generally struggle to keep up with conversation,” “severe hearing loss; often rely on lip reading as well as hearing”). The primary cause of tinnitus was scored according to the judgement of the participant, as well as if tinnitus caused any health-related conditions according to their opinion [e.g., stress, anxiety, depression, insomnia, panic attacks, depression, concentration problems, obsessive compulsive disorder (OCD-) type conditions, and substance abuse]. To score the effect of tinnitus on daily life, the awareness, bothersomeness, and frustration of the tinnitus over the last week was recorded on a scale from 1 to 10 (1 = not at all; 10 = constantly). The effect of psychological (stress, anxiety, depression, panic attacks, and OCD-like conditions) and physical factors (jaw and neck problems, physical disabilities) were scored for their influence on the experienced tinnitus (made a lot better, made a little better, no difference, made a little worse, made a lot worse).

The participants were asked if they underwent any treatments for their tinnitus. If yes, items out of a long list of potential therapies were asked to be checked when applicable (consisting of pharmacological-, psychological-, and sound-therapy treatments and non-conventional therapies). Secondly, the effect of these therapies on tinnitus was rated by the participant (big improvement, small improvement, no change, made me slightly worse, made me a lot worse).

### Outcome Measures: Survey Physical Links

The second questionnaire consisted of 22 questions and aimed at assessing the factors of influence on the experienced tinnitus. Questions were encompassing demographic variables, as well as the impact of tinnitus on daily life, tinnitus characteristics and cause, comorbid conditions (in particular, somatic factors), and the influence of factors on tinnitus (Attachment 2 Survey “Physical Links”).

Demographic variables included gender and age in years. The impact of tinnitus was assessed as loudness and annoyance of tinnitus over the past week on a scale from 0 to 10 (loudness: 0 = do not hear, 10 = extremely loud/annoyance: 0 = never annoyed, 10 = constantly annoyed). The participants rated the last week awareness on a scale from 0 to 100 (0 = never aware, 100 = always aware). The primary cause of the tinnitus according to the participant was scored. Tinnitus characteristics entailed the tinnitus duration (in years), pattern of perceived sounds during the day, and the effect of sounds on tinnitus. Hearing loss was scored by the question “do you have any hearing loss?” for which the answers were divided into categories as mentioned for survey “Treatment for tinnitus” with the addition of two categories: “hearing loss not diagnosed but I think so” and “not diagnosed but I don't think so.”

Comorbid conditions and their origin were scored; headaches (yes; they feel like they come from the neck/jaw/neck and jaw/can't pinpoint the cause, no more than I believe is normal), grinding or clenching of the teeth, experienced pain/discomfort in the jaw, the existence of stiff or sore neck muscles, and fullness in the ears. The effect of conditions on the experienced tinnitus (e.g., psychological factors, movements, and activities) was scored in five categories, ranging from “made it a lot worse” to “made is a lot better.”

### Statistical Analysis

Descriptive analysis was used to assess the characteristics of participants and experienced tinnitus using means and SDs. Univariable logistic regression was used to calculate the relative odds of undergoing treatment or not and the effect of treatment by the different variables. Univariate linear regression was used to calculate the effect of physical conditions on awareness, loudness, and annoyance. All the data was analyzed using the R-statistics (www.R-project.org, R Core team 2013, Vienna, Austria) and the SPSS software (version 25, IBM Corp., Armonk, NY, USA). A statistically significant result will be defined as *p* < 0.05.

## Results

### Survey “Treatment for Tinnitus”

#### Characteristics of Cohort

A total of 5,017 questionnaires were completed. The majority of participants were men (*n* = 2,852; 56.8%). A total of 2,555 (50.9 %) participants were in the age range of 45–64 years of age and 1,520 (30.2%) were between 18 and 44 years of age. A total of 52 (1%) participants were under the age of 18 years (and 16 preferred not to say their age; 0.3%) (Table 1). In the majority of cases, the primary cause of tinnitus was scored as unknown (*n* = 1,440; 28.7%). Noise-induced hearing loss (*n* = 689, 13.7%) and acoustic trauma (*n* = 481, 9.6%) were scored as second and third most common causes. Most participants experienced some form of hyperacusis (*n* = 2,924, 58.3%) and reported to have some degree of hearing loss ranging from mild to severe (*n* = 3,180, 63.4%).

**Table 1:** Baseline characteristics of participants to surveys in numbers of participants (and %).

	**Survey causes and treatment (*n* = 5,017)**	**Survey physical links (*n* = 6,115)**
	***N*** **(%)**	***N*** **(%)**
**Age (yrs)**		54.1 (13.8)^*^
<18 years	52 (1.0)	
18–44 years	1,520 (30.2)	
45–64 years	2,555 (50.9)	
>65 years	874 (17.4)	
Prefer not to say	16 (0.3)	
**Gender**		
Male	2,852 (56.8)	3,154 (51.6)
Female	2,132 (42.5)	2,925 (47.8)
Transgender	17 (0.3)	17 (0.3)
Prefer not to say	16 (0.3)	19 (0.3)
**Onset of tinnitus**		
<3 months	286 (5.7)	
4–6 months	257 (5.1)	
>6–12 months	451 (9.0)	
>1–5 years	1,777 (35.4)	
>5–10 years	821 (16.4)	
>10–20 years	688 (13.7)	
>20 years	737 (14.7)	
Tinnitus duration (yrs)		4.4 (9.2)^*^
**Primary cause**		
Don't know	1,440 (28.7)	1,618 (27.6)
Virus	384 (7.7)	476 (8.1)
Ototoxic	229 (4.6)	304 (5.2)
Acoustic trauma	481 (9.6)	624 (10.7)
Age-related HL	144 (2.9)	250 (4.3)
Otosclerosis	30 (0.6)	48 (0.8)
Noise-induced HL	689 (13.7)	748 (12.8)
Barotrauma	33 (0.7)	65 (1.1)
Sudden HL	120 (2.4)	237 (4.0)
Allergy	20 (0.4)	28 (0.4)
Psychological	243 (4.8)	269 (0.5)
TMJ	74 (1.5)	141(2.4)
Dental treatment	34 (0.7)	51 (0.09)
Head or neck injury	140 (2.8)	217 (3.7)
Meniere's	116 (2.3)	170 (2.9)
Ear wax procedure	50 (1.0)	69 (1.1)
Ear wax build up	31 (0.6)	36 (0.6)
Metabolic	19 (0.4)	55 (0.9)
Spontaneous onset	322 (6.4)	–
Other	418 (8.3)	340 (5.8)
Eustachian tube dysfunction	–	108 (1.8)
Missing	–	261
**Hyperacusis**		
No	1,893 (37.7)	
Mildly	1,301 (25.9)	
Moderately	1,167 (23.3)	
Severely	456 (9.1)	
Don't know	200 (4.0)	
**Pulsatile tinnitus**		
Yes	889 (17.7)	630 (10.3)
No	3,651 (72.8)	5,485 (89.7)
**Somatic tinnitus**		
Yes	1,647 (32.8)	
No	2,950 (58.8)	
**Hearing loss**		
No	1,837 (36.6)	1,219 (19.9)
Mild HL	2,139 (42.6)	2,071 (33.9)
Moderate HL	742 (14.8)	1,380 (22.6)
Severe HL	299 (6.0)	383 (6.3)
Not diagnosed, but think so		522 (8.5)
Not diagnosed, but don't think so		540 (8.8)
Tinnitus awareness^*^ (0–10)	8.6 (2.2)	75.4 (27.6)^#^
Tinnitus bothering^*^ (0–10)	6.7 (3.0)	
Tinnitus frustration^*^ (0–10)	5.7 (3.1)	
Tinnitus loudness^*^ (0–10)		6.9 (2.2)
Tinnitus annoyance^*^ (0–10)		6 (3.0)
**Tinnitus caused**		
Stress	2,479 (49.4)	
Anxiety	2,332 (46.5)	
Panic-attacks	758 (15.1)	
Depression	1,697 (33.8)	
Concentration problems	2,179 (43.4)	
OCD type condition	189 (3.8)	
Insomnia	1,955 (39.0)	
Alcohol abuse	175 (3.5)	
Drug abuse	72 (1.4)	
Other	356 (7.1)	
**Tinnitus pattern**		
No changes in volume		1,860 (30.4)
Grows louder during day		1,201 (19.6)
Gets quieter during day		351 (5.7)
Changes without pattern		2,703 (44.2)
**Effect of sounds on tinnitus**		
Some make it worse		2,285 (37.4)
Some worse, some better		950 (15.5)
Some make it better		645 (10.5)
No difference		2,235 (36.5)
**Tinnitus louder by movement**		
Pressing jaw to the side		902 (14.7)
Pressing jaw backwards		858 (14.0)
Pressing jaw outwards		1,304 (21.3)
Pushing against forehead		1,090 (17.8)
Clenching teeth		1,419 (23.2)
Tilting head backwards		903 (14.8)
No change head /jaw movement		3,437 (56.2)

* *When indicated with mean (SD) are given*.

# *Score on a scale 0–100 instead of 0–10*.

*HL, hearing loss*.

#### Tinnitus Treatment and Effect

Of the 5,017 participants in this survey, 2,914 (58.1%) used one or more therapies (range 1–21) currently or in the past with a median of three treatments per participant. Of the 2,914 treated patients, 1,755 (60.2%) reported the use of 1–3 different listed treatments, 771 (26.3%) 4–6 treatments, and 388 (13.3%) even ≥7 treatments. Of those using therapy, 1,562 (31.1%) used self-administered sound therapy, 1,157 (23.1%) used supplements/herbal medicines, 785 (15.6%) used antidepressants, 621 (12.4%) used acupuncture, and 641 (12.8%) used other treatments not listed. A total of 681 (13.8%) participants reported using a hearing aid and 503 (10.0%) used an in-ear masker (Table 2). Only 371 (7%) participants used CBT, with a similar number using Tinnitus Retraining Therapy (TRT) (*n* = 370; 7.4%).

**Table 2:** Numbers of participants which underwent a treatment out of survey “Causes and Treatment” (*n* = 5,017) with outcomes per treatment [numbers according to scored outcome per treatment and (%)].

**Therapy type**	**Participants which used therapy** *N* **(%)**	**Big improvement** *N* **(%)**	**Small improvement** *N* **(%)**	**No change *N* (%)**	**Made me slightly worse** *N* **(%)**	**Made me a lot worse** *N* **(%)**
TRT	370 (7.4)	50 (13.5)	134 (36.2)	164 (44.3)	16 (4.3)	6 (1.6)
In-ear masker	503 (10.0)	34 (6.8)	199 (39.6)	227 (45.1)	32 (6.4)	11 (2.2)
CBT	371 (7.4)	46 (12.4)	158 (42.6)	159 (42.9)	5 (1.3)	3 (0.8)
Psychiatrist	298 (5.9)	18 (6.0)	89 (29.9)	174 (58.4)	8 (2.7)	9 (3.0)
Psychologist	388 (7.7)	36 (9.3)	137 (35.3)	201 (51.8)	9 (2.3)	5 (1.3)
Neuromonics	95 (1.9)	6 (6.3)	20 (21.1)	62 (65.3)	4 (4.2)	3 (3.2)
SoundCure	144 (2.9)	8 (5.6)	43 (29.9)	80 (55.6)	7 (4.9)	6 (4.2)
Acoustic neuromodulation	120 (2.4)	7 (5.8)	37 (30.8)	73 (60.8)	2 (1.7)	1 (0.8)
Notched music therapy	223 (4.4)	4 (1.8)	64 (28.7)	145 (65.0)	10 (4.5)	0 (0.0)
Hearing aid	681 (13.6)	98 (14.4)	276 (40.5)	236 (34.7)	44 (6.5)	27 (4.0)
Self-administered sound therapy	1,562 (31.1)	146 (9.3)	798 (51.1)	584 (37.4)	26 (1.7)	8 (0.5)
Bio or neuro feedback / meditation	270 (5.4)	21 (7.8)	112 (41.5)	131 (48.5)	5 (1.9)	1 (0.4)
Antidepressants	785 (15.6)	64 (8.2)	280 (35.7)	340 (43.3)	68 (8.7)	33 (4.2)
GABA type drugs	237 (4.7)	23 (9.7)	86 (36.3)	104 (43.9)	16 (6.8)	8 (3.4)
Retigabine	53 (1.1)	15 (28.3)	18 (34.0)	16 (30.2)	2 (3.8)	2 (3.8)
Transcranial stimulation	45 (0.9)	2 (4.4)	8 (17.8)	31 (68.9)	2 (4.4)	2 (4.4)
HBOT	46 (0.9)	2 (4.3)	11 (23.9)	32 (69.6)	1 (2.2)	0 (0.0)
Steroids	346 (6.9)	23 (6.6)	82 (23.7)	218 (63.0)	11 (3.2)	12 (3.5)
Low-level laser treatment	65 (1.3)	4 (6.2)	11 (16.9)	42 (64.6)	7 (10.8)	1 (1.5)
Off-label medication	312 (6.2)	9 (2.9)	76 (27.2)	217 (69.6)	7 (2.2)	3 (1.0)
Surgical procedure	36 (0.7)	1 (2.8)	8 (22.2)	17 (47.2)	3 (8.3)	7 (19.4)
Acupuncture	621 (12.4)	13 (2.1)	148 (23.8)	445 (71.7)	12 (1.9)	3 (0.5)
Chiropractor	489 (9.7)	11 (2.2)	97 (19.8)	367 (75.1)	10 (2.0)	4 (0.8)
Supplements / herbal medicines	1,157 (23.1)	32 (2.8)	247 (21.3)	860 (74.3)	12 (1.0)	6 (0.5)
Tinnitus cure eBooks	254 (5.1)	8 (3.1)	53 (20.9)	189 (74.4)	2 (0.8)	2 (0.8)
Homeopathic treatment	425 (8.5)	13 (3.1)	79 (18.6)	323 (76.0)	6 (1.4)	4 (0.9)
Other treatment	641 (12.8)	130(20.3)	224 (34.9)	261 (40.7)	14 (2.2)	12 (1.9)

For the majority of therapies, most participants reported that there were no changes due to the therapy (Table 2). Only in case of hearing aids, self-administered sound therapy, and retigabine usage, the majority of participants noted a small improvement due to the treatment. Overall, only a small number of participants reported any degree of worsening caused by the experienced therapy, except for surgical treatments (treatment not specified). For the latter, in seven out of 36 cases (19.4%), this was scored as “made it a lot worse.”

#### Comparison of Participants With and Without Therapy

Table 3 shows the outcomes for the comparison of participants using tinnitus therapy compared to those not using it. Being female [odds ratio (OR) 0.83 (95% CI 0.74–0.93, *p* < 0.01)], having mild [OR 1.43 (95% CI 1.12–1.83, *p* < 0.01)] or moderate hearing loss [OR 1.40 (96% CI 1.10–1.78, *p* < 0.01)], and moderate [OR 1.70 (95% CI 1.26–2.30, *p* < 0.01)] or severe [OR 1.78 (95% CI 1.32–2.41, *p* < 0.01)] hyperacusis were statistically significantly associated with higher odds of using (or having used) tinnitus treatment. Having higher tinnitus impact on daily life awareness [OR 1.05 (95% CI 1.02–1.08, *p* < 0.01)], bothersomeness [OR 1.08 (95% CI 1.06–1.10, *p* < 0.01)] or frustration [OR 1.12 (96% CI 1.10–1.14, *p* < 0.01)], or experiencing physical and psychological effects by the tinnitus were all significantly statistically associated with higher odds of having tinnitus treatment (Table 3).

**Table 3:** Outcomes of univariate regression analysis of tinnitus impact and characteristics and psychological/ physical effects of tinnitus between those with and without having treatment (survey “Treatment for tinnitus”).

**Comparison of participants with and without therapy**			
	**Treatment**	**No treatment**	**OR**
	**(*****n*** **=** **2,914)**	**(*****n*** **=** **2,103)**	**(95% CI**, ***p*****-value)**
	***N*** **(%)**	***N*** **(%)**	
**Gender**			
Male	1,712 (58.8)	1,140 (54.2)	Ref
Female	1,181 (40.5)	951 (45.2)	0.83 (0.74–0.93, *p* <0.01)
Transgender	11 (0.4)	6 (0.3)	1.22 (0.46–3.55, *p* = 0.70)
Prefer not to say	10 (0.3)	6 (0.3)	1.11 (0.41–3.27, *p* = 0.84)
**Hearing loss**			
None	1,097 (37.6)	740 (32.5)	Ref
Mild	1,265 (43.4)	874 (41.6)	1.43 (1.12–1.83, *p* <0.01)
Moderate	400 (13.7)	342 (16.3)	1.40 (1.10–1.78, *p* <0.01)
Severe	152 (5.2)	147 (7.0)	1.13 (0.87–1.48, *p* = 0.37)
**Hyperacusis**			
No	1,006 (34.5)	887 (42.2)	Ref
Mild	795 (27.3)	506 (24.1)	1.23 (0.92–1.65, *p* = 0.10)
Moderate	726 (24.9)	441 (21.0)	1.70 (1.26–2.30, *p* <0.01)
Severe	291 (10.0)	165 (7.8)	1.78 (1.32–2.41, *p* <0.01)
Don't know	96 (3.3)	104 (4.9)	1.91 (1.36–2.68, *p* <0.01)
Tinnitus awareness	8.7 (2.1)	8.5 (2.3)	1.05 (1.02–1.08, *p* <0.01)
Tinnitus bothering	6.9 (2.9)	6.3 (3.0)	1.08 (1.06–1.10, *p* <0.01)
Tinnitus frustration	6.1 (3.1)	5.0 (3.1)	1.12 (1.10–1.14, *p* <0.01)
**Pulsatile tinnitus**			
No	488 (16.7)	401 (19.1)	Ref
Yes	2,168 (74.4)	1,483 (70.5)	1.03 (0.83–1.30, *p* = 0.78)
Unsure	258 (8.9)	219 (10.4)	1.24 (1.02–1.50, *p* = 0.03)
**Somatic tinnitus**			
No	1,056 (36.2)	591 (28.1)	Ref
Yes	1,643 (56.4)	1,307 (62.1)	1.70 (1.37–2.12, *p* <0.01)
Unsure	215 (7.4)	205 (9.7)	1.20 (0.98–1.47, *p* = 0.08)
**Tinnitus caused me…**.			
**Stress**			3.09 (2.75–3.47, *p* <0.01)
Yes	1,140 (55.9)	1,398 (55.1)	
No	1,774 (71.6)	705 (28.4)	
**Anxiety**			2.94 (2.62–3.31, *p* <0.01)
Yes	1,242 (46.3)	1,443 (53.7)	
No	1,672 (71.7)	660 (28.3)	
**Panic attacks**			2.85 (2.38–3.42, *p* <0.01)
Yes	2,327 (54.6)	1,932 (45.4)	
No	587 (77.4)	171 (22.6)	
**Depression**			3.31 (2.91–3.77, *p* <0.01)
Yes	1,624 (48.9)	1,696 (51.1)	
No	1,290 (76.0)	407 (24.0)	
**OCD type condition**			1.79 (1.31–2.49, *p* <0.01)
Yes	2,780 (57.6)	2,048 (42.4)	
No	134 (70.9)	55 (29.1)	
**Insomnia**			2.66 (2.36–3.00, *p* <0.01)
Yes	1,506 (49.2)	1,556 (50.8)	
No	1,408 (72.0)	547 (28.0)	
**Concentration/focus problems**			2.53 (2.25–2.85, *p* <0.01)
Yes	1,378 (48.6)	1,460 (51.4)	
No	1,536 (70.5)	643 (29.5)	
**Alcohol abuse**			2.78 (1.94–4.06, *p* <0.01)
Yes	2,776 (57.3)	2,066 (42.7)	
No	138 (78.9)	37 (21.1)	
**Drug abuse**			1.89 (1.15–3.25, *p* = 0.02)
Yes	2,862 (57.9)	2,083 (42.1)	
No	52 (72.2)	20 (27.8)	

*Ref, reference category*.

### Survey Physical Links

#### Characteristics of Cohort

In total, 6,115 participants completed the survey “Physical Links” (Table 1). The majority of participants were men [*n* = 3,154 (51.6%)]. Mean age of the participants was 54.1 years (SD 13.8). Similar to survey one, the most reported primary cause of tinnitus was unknown (*n* = 1,618, 27.6%), noise induced hearing loss (*n* = 748, 12.8%), and acoustic trauma (*n* = 624, 10.7%). The majority of cases had some (diagnosed or self-reported) degree of hearing loss (*n* = 4,356, 71.2%).

#### Physical and Psychological Factors of Influence on the Experienced Tinnitus

The largest percentage of responders indicated that sleep (good sleep, bad sleep, or napping) made no difference in tinnitus experience [respectively, in 2,304 (37.7%), 1,875 (30.7%), and 2,668 (43.6%) of cases] (Table 4). Physical exercise made no difference in tinnitus experience for the majority of participants [intense work-out (2,078, 34.0%), moderate (2,496, 40.8%), or light exercise (3,510, 57.4%)]. Both anxiety and stress were mostly scored as making tinnitus a little worse [respectively, in 2,094 (34.2%) and 2,167 (35.4 %) of cases] or a lot worse [respectively, in 1,662 (27.2%) and 1,809 (29.6%)]. Specific movements of the jaw, head, and teeth made the tinnitus louder in 14.0–23.2% of cases depending on the type, whereby about half of the respondents experienced no change to their tinnitus caused by movement of the head or jaw (*n* = 3,437, 56.2%) (Table 1).

**Table 4:** Effect of physical and psychological factors on experienced tinnitus out of survey physical links (*n* = 6,115).

**“…..made the tinnitus..”**		**A lot worse**	**A little worse**	**No difference**	**A little better**	**A lot better**	**Unsure/don't know**
		***N*** **(%)**	***N*** **(%)**	***N*** **(%)**	***N*** **(%)**	***N*** **(%)**	***N*** **(%)**
Sleep	Good sleep	303 (5)	469 (7.7)	2,304 (37.7)	1,471 (24.1)	710 (11.6)	858 (14.0)
	Bad sleep	1,406 (23.0)	1,951 (31.9)	1,875 (30.7)	53 (0.9)	17 (0.3)	813 (13.3)
	Napping	572 (9.4)	832 (13.6)	2,668 (43.6)	453 (7.4)	54 (0.9)	1,536 (25.1)
Exercise	Intense workout	351 (5.7)	844 (13.8)	2,078 (34.0)	385 (6.3)	111 (1.8)	2,346 (38.4)
	Moderate exercise	223 (3.6)	901 (14.7)	2,496 (40.8)	588 (9.6)	116 (1.9)	1,791 (29.3)
	Light exercise	80 (1.3)	494 (8.1)	3,510 (57.4)	764 (12.5)	138 (2.3)	1,129 (18.5)
Psychological	Anxiety	1,662 (27.2)	2,094 (34.2)	1,294 (21.2)	23 (0.4)	10 (0.2)	1,032 (16.9)
	Stress	1,809 (29.6)	2,167 (35.4)	1,186 (19.4)	21 (0.30)	6 (0.10)	926 (15.1)

*Frequencies and (%)*.

#### Characteristics Related to the Experienced Tinnitus

Table 5 shows the outcomes for the univariate regression analysis for the effect of demographics, etiology, and physical factors and comorbidities on the experienced tinnitus during the past week. Statistically significant outcomes are described. Women experienced a statistically significant higher tinnitus awareness (β 5.36, 95% CI 3.98–6.75, *p* < 0.01), annoyance (β 0.54, 95% CI 0.39–0.68; *p* < 0.01), and loudness (β 0.38, 95% CI 0.27–0.49, *p* < 0.01) compared to men. Overall, sudden hearing loss, head or neck injury, Meniere's disease, headaches, fullness in the ears, and all categories of hearing loss were significantly positively associated with the experienced tinnitus loudness, annoyance, and awareness. Other factors showed statistically inconsistent relations compared to the categories of tinnitus impact.

**Table 5:** Outcome of univariate regression analysis of effect of demographics, etiology, and physical factors/comorbidities on experienced impact of tinnitus during the past week (survey physical links).

		**Tinnitus loudness**			**Tinnitus annoyance**			**Tinnitus awareness**		
	***N***	**Mean (SD)**	**B (95%CI)**	***P***	**Mean (SD)**	**B (95%CI)**	***P***	**Mean (SD)**	**B (95%CI)**	***P***
**Age**		7.0 (2.2)	0.03 (0.02–0.03)	**<0.01**	6.9 (3.0)	0.02 (0.01–0.02)	**<0.01**	68.5 (27.6)	0.23 (0.27–0.36)	**<0.01**
**Sex**										
Male	3,139	6.8 (2.2)	Ref	–	6.6 (2.9)	Ref	–	66.9 (28.2)	Ref	–
Female	2,918	7.2 (2.3)	0.38 (0.27–0.49)	**<0.01**	7.1 (3.0)	0.54 (0.39– 0.68)	**<0.01**	71.3 (26.8)	5.36 (3.98–6.75)	**<0.01**
Transgender	17	7.3 (2.6)	0.52 (−0.53–1.57)	0.33	7.9 (2.9)	1.33 (−0.08 −2.74)	0.06	75.1 (32.1)	9.18 (−3.94–22.30)	0.17
Prefer not to say	19	7.8 (2.1)	1.01 (0.02–2.01)	0.05	7.3 (3.3)	0.65 (−0.68– 1.98)	0.34	71.6 (27.6)	5.65(−6.77–18.06)	0.37
**Etiology**										
Unknown	1,618	6.9 (2.1)	Ref	–	6.8 (3.0)	Ref	-	68.13 (28.24)	Ref	-
Sudden HL	237	7.5 (2.2)	0.58 (0.28–0.89)	**<0.01**	7.6 (3.1)	0.84 (0.44– 1.24)	**<0.01**	74.65 (27.83)	6.52 (2.76–10.27)	**0.01**
Noise induced HL	748	7.0 (2.1)	0.14 (−0.5–0.33)	0.16	6.7 (2.9)	−0.04 (−0.30–0.21)	0.75	66.56 (27.50)	−1.57 (−3.96–0.81)	0.20
Noise trauma	624	6.8 (2.2)	−0.7 (−0.27–0.13)	0.50	6.8 (2.8)	0.04 (−0.24–0.31)	0.79	65.25 (27.70)	−2.87 (−5.42–−0.33)	**0.03**
Virus	476	6.6 (2.2)	−0.27 (−0.50–−0.05)	**0.02**	6.4 (2.9)	−0.37 (−0.67-−0.07)	**0.02**	65.78 (27.49)	−2.35 (−5.16–0.47)	0.10
Ototoxic	304	6.9 (2.2)	0.03 (−0.24–0.30)	0.84	7.0 (2.8)	0.28 (−0.08–0.64)	0.13	69.48 (27.30)	1.35 (−2.3–4.72)	0.43
Age related HL	250	6.5 (2.0)	−0.36 (−0.62–−0.07)	**0.02**	6.2 (2.9)	−0.53 (−0.93-−0.14)	**0.01**	65.64 (28.13)	−2.49 (−6.16–1.18)	1.87
Eustachian tube dysfunction	108	6.9 (2.3)	−0.02 (−0.45–0.41)	0.94	7.4 (3.1)	0.62 (0.05–1.19)	**0.03**	71.80 (26.83)	3.67 (−1.70–9.03)	0.18
Otosclerosis	48	7.3 (2.2)	0.43 (−0.21–1.06)	0.19	6.8 (3.0)	0.02 (−0.82 −0.87)	0.96	74.56 (25.68)	6.43 (−1.47–14.34)	0.11
Barotrauma	65	7.3 (2.4)	0.36 (−0.19–0.90)	0.20	6.9 (2.8)	0.12 (−0.61– 0.85)	0.74	69.86 (28.23)	1.73 (−5.09–8.56)	0.62
Allergy	28	6.5 (2.1)	−0.43 (−1.26–0.41)	0.32	6.6 (2.8)	−0.16 (−1.26–0.94)	0.78	66.00 (27.64)	−2.13 (−12.41–8.16)	0.69
Psychosocial	269	6.8 (2.4)	−0.15 (−0.43–0.14)	0.31	6.9 (3.2)	0.12 (−0.26–0.51)	0.52	66.29 (28.57)	−1.84 (−5.39–1.72)	0.31
TMJ	141	6.5 (2.2)	−0.41 (−0.79–−0.03)	**0.04**	6.9 (2.8)	0.10 (−0.41–0.61)	0.69	66.58 (26.77)	−1.55 (−6.29–3.19)	0.52
Dental treatment	51	7.0 (2.2)	0.07 (−0.54–0.69)	0.81	6.8 (3.1)	0.02 (−0.80–0.84)	0.97	66.86 (26.86)	−1.27 (−8.94–6.41)	0.75
Head or neck injury	217	7.6 (2.4)	0.71 (0.40–1.03)	**<0.01**	**7.9 (2.8)**	**1.12 (0.70–1.53)**	**<0.01**	**74.66 (24.93)**	**6.54 (2.63–10.44)**	**<0.01**
Meniere's	170	7.5 (2.4)	0.58 (0.23–0.92)	**<0.01**	**7.3 (2.9)**	**0.51 (0.04–0.97)**	**0.03**	**74.08 (27.04)**	**5.95 (1.60–10.30)**	**0.03**
Earwax procedure	69	7.1 (2.3)	0.18 (−0.35–0.72)	0.50	7.7 (2.8)	0.91 (0.20–1.62)	**0.01**	**75.59 (24.37)**	**7.47 (0.83–14.10)**	**0.03**
Earwax build up	36	5.8 (2.3)	−1.10 (−1.83–−0.37)	**0.01**	6.6 (2.9)	−0.21 (−1.18–0.76)	0.67	58.58 (29.26)	−9.55 (−18.64–−0.45)	**0.04**
Metabolic	55	6.9 (2.0)	0.04 (−0.55–0.63)	0.90	6.4 (2.8)	−0.39 (−1.18–0.41)	0.34	65.95 (22.69)	−2.18 (−9.58–5.21)	0.56
Not listed	340	7.3 (2.3)	0.40 (0.14–0.66)	**<0.01**	**7.2 (3.1)**	**0.43 (0.09–0.77)**	**0.02**	**74.41 (26.14)**	**6.28 (3.06–9.50)**	**<0.01**
**Stiff/sore neck muscles**			−0.11 (−0.36–0.14)	0.38		−0.15 (−0.48–0.18)	**<0.01**		−2.17 (−5.25–0.91)	0.17
No	3,268	7.1 (2.2)			7.0 (2.9)			70.6 (25.8)		
Yes	5,787	7.0 (2.2)			6.9 (3.0)			68.4 (27.7)		
**Headaches**			0.43 (0.31–0.54)	**<0.01**		0.83 (0.68–0.98)	**<0.01**		6.15 (4.73–7.57)	**<0.01**
No	3,790	6.8 (2.2)			6.6 (2.9)			66.2 (28.2)		
Yes	2,325	7.2 (2.2)			7.4(2.9)			72.4 (26.3)		
**Fullness in the ears**			0.37 (0.25–0.48)	**<0.01**		0.69 (0.54–0.84)	**<0.01**		5.89 (4.48–7.29)	**<0.01**
No	2,460	6.7 (2.2)			6.5 (3.0)			65.0 (28.6)		
Yes	3,655	7.1 (2.2)			7.2 (2.9)			70.9 (26.7)		
**Pain/discomfort jaw**			0.19 (−0.04–0.20)	0.18		0.34 (0.18–0.49)	**<0.01**		1.91 (0.46–3.36)	**0.01**
No	3,949	6.9 (2.2)			6.8 (3.0)			67.9 (28.0)		
Yes	2,166	7.0 (2.2)			7.1 (2.9)			69.8 (26.9)		
**Hearing loss**										
No	1,219	6.4 (2.3)	Ref	–	6.5 (3.0)	Ref	–	65.3 (28.2)	Ref	–
Mild HL	2,071	6.9 (2.2)	0.46 (0.31–0.62)	**<0.01**	6.8 (2.9)	0.30 (0.09–0.51)	**<0.01**	67.7 (27.3)	2.43 (0.50–4.35)	**0.01**
Moderate HL	3,803	7.5 (2.1)	1.08 (0.92–1.25)	**<0.01**	7.3 (2.9)	0.74 (0.52–0.97)	**<0.01**	73.6 (25.7)	8.29 (6.20–10.39)	**<0.01**
Severe HL	83	8.2 (2.3)	1.71 (1.47–1.96)	**<0.01**	8.1 (2.9)	1.57 (1.23–1.90)	**<0.01**	80.8 (26.0)	15.53 (12.40–18.65)	**<0.01**
Not diagnosed, but think so	522	6.7 (2.1)	0.29 (0.07–0.51)	**0.01**	6.6 (2.9)	0.09 (−0.21–0.40)	0.53	66.5 (27.7)	1.26 (−1.53–4.04)	0.38
Not diagnosed, but don't	540	6.2 (2.2)	−0.23 (−0.44–0.01)	**0.04**	6.1 (3.0)	−0.40 (−0.69–−0.10)	**<0.01**	59.7 (28.8)	−5.58 (−8.34–−2.82)	**<0.01**
think so									

*Ref, reference category; HL, hearing Loss*.

*In bold outcomes with a statistically significant result defined as a p-value < 0.05*.

## Discussion

Most of the current knowledge about people with tinnitus and treatment outcomes is retrieved from studies examining a sample of patients visiting tinnitus health care centers for diagnosis, therapy, or counseling. Considering the lack of curative treatments, it is not surprising that people with tinnitus keep looking for symptom relief with the help of complementary or alternative medicine (14). By assessing the characteristics and effect of therapy of people with tinnitus responding to a worldwide internet-based survey, we were able to gain insight in a group of people with tinnitus not necessarily visiting a health care center for their tinnitus. Thereby, we have to keep in mind that these results do reflect thoughts and assumptions of participants about the cause of tinnitus, tinnitus-related difficulties, and experienced treatment effects.

Half of our patients used more than one therapy. Of those using treatment, about 2 out of 5 people used > 4 tinnitus therapies, including conventional and non-conventional tinnitus therapies. This number might not be representative for the average tinnitus patient, but do reflect a self-selecting group of people who struggle with their tinnitus enough to seek help online and willing to respond to a survey posted on Tinnitus Talk. Thereby, someone who is not bothered at all by their tinnitus is unlikely to seek out online support groups and would not notice the survey requests. Besides this, it is possible that patients with more help-seeking behavior are more prone to fill out the online questionnaires. Still, this outcome is of highly interest as it reflects the ongoing search for tinnitus relief in people bothered by their tinnitus. These people who are struggling are the ones who need help and better treatments, thus exactly the subgroup of interest for researchers and clinicians for future improvements of tinnitus health care.

Being female, having higher tinnitus impact on daily life, and experiencing physical or psychological effects by the tinnitus were all associated with statistically significant higher odds of using tinnitus treatment. Self-administered sound therapy, supplements/herbal medicines, antidepressants, and acupuncture were most commonly used. This can be related to the costs or accessibility of treatments. Most (sub)types of listed sound therapies, psychological therapies, as well as drug therapies (such as antidepressants, GABA-type drugs, and retigabine) were rated as having a positive effect on their tinnitus by the reports of the participants. This is of interest considering the lack of positive effects to tinnitus found in systematic reviews so far, assessing most of these therapies applied in clinical studies, or the lack of evidence for others (10, 11, 15). Currently, by its proven effectiveness, cognitive behavior therapy is advocated to treat tinnitus-related distress in those whose tinnitus is causing an impact on their well-being and activities (10, 16). In our study, 55% of people who tried CBT experienced a degree of improvement, where about 40% reported to have no change to their tinnitus. Although, to explain the positive results on many therapies reported by participants, we need to keep in mind that the expericend effect can be influenced by a placebo effect as described for several tinnitus therapies (17). Besides this, many participants have undergone multiple treatments by which it can be difficult to recall and assess the effect of a specific (simultaneous or sequential) therapy (many years) afterwards. On the other hand, the order effect (the order in which the participant received a therapy may affect the outcome), measurement (or “judgement”) errors of repeated individual evaluations, and the classification of therapy outcomes into categories of effect could distort the real effect of therapies. Lastly, out of this survey, we were not able to verify if tinnitus treatments were used by the stated indication criteria or were provided according to treatment standards (such as for psychological treatments), which could influence the experienced effectiveness. To sort these effects out, influencing outcomes of (multiple) therapies, prospective, protocollized, and, when possible, randomized controlled studies would be needed. Considering the possible placebo effect of tinnitus therapies, it would be beneficial to describe these effects in more detail.

Anxiety and stress, as well as being female, having a higher age, certain listed causes, experiencing headaches, fullness in the ears, and any degree of hearing loss were statistically significant associated with a higher experienced tinnitus loudness, annoyance, or awareness. However, the latter effect sizes were mostly <1 point difference on loudness and annoyance scores (both on a scale from 0 to 10) or < 10 points change on awareness scores (on a scale from 0 to 100) and therefore not deemed to be clinically relevant. From the few previous reports examining the relationship between the impact of tinnitus and tinnitus (un)related characteristics, the study from Hiller and Goebel is worthwhile to mention. In a similar non-clinical sample of members of the German Tinnitus League, they demonstrated that tinnitus loudness and annoyance were higher in subjects with hearing loss, vertigo, and hyperacusis (18). Secondly, substantially higher rates of loudness and annoyance were found in those whose tinnitus was due to conductive hearing loss, severe head injury, or neurologic disease (18). But in this study, the degrees of tinnitus loudness and annoyance were categorized in grades of severity, hindering comparison of effect-sizes with outcomes of our study.

The strength of this study is the worldwide cohort, in which participants were not necessarily related to a specific health care center or clinic. Nonetheless, several limitations need to be addressed. First of all, recall bias could have been introduced by the fact that in both surveys, people were asked to answer questions about their previous experiences with factors of influence on their tinnitus and the effect of tinnitus treatments. Secondly, outcomes of the study rely on data of two surveys which could be completed by the same participants. Thirdly, the survey was written in English language which could have introduced limitations for people to attend the survey and limits the generalizability of outcomes. Fourthly, the presence or absence of symptoms or diseases was scored according to the subjective judgement of the participant by which outcomes could deviate from objective measures such as for hearing loss. Next, survey questions on diseases and symptoms such as the question phrased to assess hyperacusis (classically defined as a negative reactions to a sound only depending on its physical characteristics) could also encompass other subdivisions of decreased sound tolerance such as misophonia (in the literature defined as an abnormally strong reaction to a sound with a specific pattern and meaning to a given subject) (19). Therefore, outcomes need to be interpreted carefully and in line with the phrased question.

Regrettably, an effective treatment for tinnitus is still not available. As demonstrated in our study, more than 50% of participants used >1 type of conventional or non-conventional therapies, in many cases without proven (and experienced) benefit. From this outcome, one could question the accessibility of up-to-date information about the effectiveness of tinnitus treatments in current tinnitus health care for patients and health care providers. Optimization of this information would necessitate combined efforts of patients, physicians, and stakeholders and could potentially reduce the consumption of (non)conventional health care for tinnitus. On the other hand, the outcomes of our study created insights about experiences, thoughts, and beliefs of people with tinnitus, which could help in optimizing tinnitus health care of those in need for help. Moreover, the high number of individuals experiencing no clinical impact of treatment on tinnitus underlines the need for finding a personalized and accessible treatment for patients with tinnitus. Future research should investigate how physical, mental health, and tinnitus-related factors influence and predict the impact of tinnitus on peoples' lives and which factors explain the experienced effect of treatment on individuals. This could result in finding “subtypes” of patients with tinnitus that respond to certain types of therapy. Collaboration between research groups to combine data sets is the first step to achieve this goal.

## Data Availability Statement

The raw data supporting the conclusions of this article will be made available by the authors, without undue reservation.

## Ethics Statement

The studies involving human participants were reviewed and approved by Ethical Committee of the University Medical Center Utrecht, Netherlands (local number: 19-134/C). The patients/participants provided their written informed consent to participate in this study.

## Author Contributions

AS wrote the manuscript text and interpreted the data. MV designed and conducted the surveys and revised the manuscript. HG revised the manuscript and assisted in drafting conclusion. JE and CW assisted in organizing the outcomes of the surveys. IS conducted the statistical analysis and assisted in writing the results. All authors reviewed the manuscript.

## Conflict of Interest

MV and HG were employed by Tinnitus Hub Ltd. The remaining authors declare that the research was conducted in the absence of any commercial or financial relationships that could be construed as a potential conflict of interest.

## Publisher's Note

All claims expressed in this article are solely those of the authors and do not necessarily represent those of their affiliated organizations, or those of the publisher, the editors and the reviewers. Any product that may be evaluated in this article, or claim that may be made by its manufacturer, is not guaranteed or endorsed by the publisher.
