# Supplementary material for: Tinnitus: Characteristics, Need for Therapy, and Therapeutic Outcomes; Results of an International Patient Initiated Platform
Source: Front Neurol. 2022 Jan 20;12:778450. doi: 10.3389/fneur.2021.778450 (PMC8810819; doi:10.3389/fneur.2021.778450)
Supplement: Supplementary file 1 [file Data_Sheet_1.PDF]

Tinnitus Hub (Tinnitus Talk) 2016

About you

*** 1. Are you...**

- Male
- Female
- Transgender
- Prefer not to say

*** 2. Please let us know your age.**

- Under 18
- 18-24
- 25-34
- 35-44
- 45-54
- 55-64
- 65-74
- 75+
- Prefer not to say

*** 3. How long have you had tinnitus for?**
(when did it first begin?)

- Less than 3 months
- 4-6 months
- 6-12 months
- 1-2 years
- 2-3 years
- 3-5 years
- 5-10 years
- 10-20 years
- 20+ years

About your tinnitus

*** 4. What is the primary cause of your tinnitus?**

(think of the main thing, what set it off when you first noticed it?)

*** 5. Can you identify a secondary cause for your tinnitus?**

- ○ Yes
- ○ No

About your tinnitus (continued)

*** 6. What is the secondary cause of your tinnitus?**

*** 7. Can you identify a tertiary (third) cause for your tinnitus?**

- Yes
- No

About your tinnitus (continued)

*** 8. What is the tertiary cause of your tinnitus?**

Noise and Somatic

*** 9. Is your tinnitus reactive to noise?**

- Mixture - some sounds make it better and some make it worse
- Some sounds make it a lot worse
- Some sounds make it a little worse
- Sounds don't really affect me
- Some sounds make it a little better
- Some sounds make it a lot better
- I don't know

*** 10. Do you have hyperacusis?**
(hyperacusis is a sensitivity to sounds, often you will think sounds are irritating and painfully loud when others hear them as normal)

- No
- Mildly
- Moderately
- Severely
- Don't know

*** 11. Do you have pulsatile tinnitus?**
(tinnitus that is rhythmic, generally in time with the heartbeat - often a whooshing or pulsing)

- Yes
- No
- Unsure

*** 12. Do you have somatic tinnitus?**
(this means you can change the volume, for example by clenching teeth or moving the jaw or neck)

- Yes
- No
- Don't know

*** 13. Do you have problems with your jaw or neck?**

(think about the past week)

- ◯ Problems with jaw
- ◯ Problems with neck
- ◯ Problems with jaw and neck
- ◯ None

Your hearing

*** 14. Do you have any hearing loss?**

- None known of
- Mild hearing loss
  (may struggle a little to keep up with conversations)
- Moderate hearing loss
  (generally struggle to keep up with conversations without a hearing aid)
- Severe hearing loss
  (often rely on lip reading as well as hearing aids to keep up with conversation)

Your hearing (continued)

*** 15. Is your hearing loss in...**

- One ear
- Both ears

*** 16. What is the main frequency range of your hearing loss?**
(phone users - scroll horizontally to see all choices)

	Unsure	Low / mid (up to 1kHz)	Mid (1kHz-3kHz)	Mid / high (4kHz-8kHz)	Very high (8kHz+)	Several dips in hearing
Frequency	◯	◯	◯	◯	◯	◯

Treatments

*** 17. Have you tried any treatments for your tinnitus?**

(think of masking, pharmacological, physical treatments as well as specialist tinnitus treatments)

- Yes
- No

Treatments (continued)

*** 18. Which treatments have you tried?**

(select all that apply)

- [ ] TRT (Tinnitus Retraining Therapy
- [ ] In-ear maskers (not as part of TRT program)
- [ ] CBT (Cognitive Behavioural Therapy)
- [ ] Psychiatrist
- [ ] Psychologist
- [ ] Neuromonics
- [ ] SoundCure
- [ ] Acoustic Neuromodulation
- [ ] Notched music therapy
- [ ] Hearing aid
- [ ] Self-administered sound therapy (masking, nature sounds)
- [ ] Bio / Neuro feedback, meditation
- [ ] Antidepressants
- [ ] GABA type drugs
- [ ] Retigabine (Trobalt / Potiga)
- [ ] Transcranial Stimulation Treatments (rTMS, tDCS, tACS)
- [ ] HBOT (Hyperbaric Oxygen Therapy)
- [ ] Steroids
- [ ] Low-Level Laser Treatment (LLLT)
- [ ] Off-label medication
- [ ] Surgical procedure
- [ ] Acupuncture
- [ ] Chiropractor
- [ ] Supplements / herbal medicines
- [ ] Tinnitus cure eBooks
- [ ] Homeopathic treatment
- [ ] Other (please specify)

Treatments (continued)

*** 19. How effective was each treatment for you?**
(phone users - scroll horizontally to see all choices)

	Big improvement	Small improvement	No change	Made me slightly worse	Made me a lot worse
TRT (Tinnitus Retraining Therapy	◯	◯	◯	◯	◯
In-ear maskers (not as part of TRT program)	◯	◯	◯	◯	◯
CBT (Cognitive Behavioural Therapy)	◯	◯	◯	◯	◯
Psychiatrist	◯	◯	◯	◯	◯
Psychologist	◯	◯	◯	◯	◯
Neuromonics	◯	◯	◯	◯	◯
SoundCure	◯	◯	◯	◯	◯
Acoustic Neuromodulation	◯	◯	◯	◯	◯
Notched music therapy	◯	◯	◯	◯	◯
Hearing aid	◯	◯	◯	◯	◯
Self-administered sound therapy (masking, nature sounds)	◯	◯	◯	◯	◯
Bio / Neuro feedback, meditation	◯	◯	◯	◯	◯
Antidepressants	◯	◯	◯	◯	◯
GABA type drugs	◯	◯	◯	◯	◯
Retigabine (Trobalt / Potiga)	◯	◯	◯	◯	◯
Transcranial Stimulation Treatments (rTMS, tDCS, tACS)	◯	◯	◯	◯	◯

	Big improvement	Small improvement	No change	Made me slightly worse	Made me a lot worse
HBOT (Hyperbaric Oxygen Therapy)	◯	◯	◯	◯	◯
Steroids	◯	◯	◯	◯	◯
Low-Level Laser Treatment (LLLT)	◯	◯	◯	◯	◯
Off-label medication	◯	◯	◯	◯	◯
Surgical procedure	◯	◯	◯	◯	◯
Acupuncture	◯	◯	◯	◯	◯
Chiropractor	◯	◯	◯	◯	◯
Supplements / herbal medicines	◯	◯	◯	◯	◯
Tinnitus cure eBooks	◯	◯	◯	◯	◯
Homeopathic treatment	◯	◯	◯	◯	◯
Other (please specify)	◯	◯	◯	◯	◯

*** 20. How long did you try each treatment for?**

(phone users - scroll horizontally to see all choices)

	Currently trying this treatment	Less than 1 month	1-2 months	2-4 months	4-6 months	More than 6 months
TRT (Tinnitus Retraining Therapy	◯	◯	◯	◯	◯	◯
In-ear maskers (not as part of TRT program)	◯	◯	◯	◯	◯	◯
CBT (Cognitive Behavioural Therapy)	◯	◯	◯	◯	◯	◯
Psychiatrist	◯	◯	◯	◯	◯	◯
Psychologist	◯	◯	◯	◯	◯	◯
Neuromonics	◯	◯	◯	◯	◯	◯
SoundCure	◯	◯	◯	◯	◯	◯
Acoustic Neuromodulation	◯	◯	◯	◯	◯	◯
Notched music therapy	◯	◯	◯	◯	◯	◯

	Currently trying this treatment	Less than 1 month	1-2 months	2-4 months	4-6 months	More than 6 months
Hearing aid	◯	◯	◯	◯	◯	◯
Self-administered sound therapy (masking, nature sounds)	◯	◯	◯	◯	◯	◯
Bio / Neuro feedback, meditation	◯	◯	◯	◯	◯	◯
Antidepressants	◯	◯	◯	◯	◯	◯
GABA type drugs	◯	◯	◯	◯	◯	◯
Retigabine (Trobalt / Potiga)	◯	◯	◯	◯	◯	◯
Transcranial Stimulation Treatments (rTMS, tDCS, tACS)	◯	◯	◯	◯	◯	◯
HBOT (Hyperbaric Oxygen Therapy)	◯	◯	◯	◯	◯	◯
Steroids	◯	◯	◯	◯	◯	◯
Low-Level Laser Treatment (LLLT)	◯	◯	◯	◯	◯	◯
Off-label medication	◯	◯	◯	◯	◯	◯
Surgical procedure	◯	◯	◯	◯	◯	◯
Acupuncture	◯	◯	◯	◯	◯	◯
Chiropractor	◯	◯	◯	◯	◯	◯
Supplements / herbal medicines	◯	◯	◯	◯	◯	◯
Tinnitus cure eBooks	◯	◯	◯	◯	◯	◯
Homeopathic treatment	◯	◯	◯	◯	◯	◯
Other (please specify)	◯	◯	◯	◯	◯	◯

*** 21. When did you last try each treatment?**

(phone users - scroll horizontally to see all choices)

	Currently undergoing this treatment	Less than 3 months ago	3-6 months ago	6-12 months ago	More than 1 year ago
TRT (Tinnitus Retraining Therapy	○	○	○	○	○
In-ear maskers (not as part of TRT program)	○	○	○	○	○
CBT (Cognitive Behavioural Therapy)	○	○	○	○	○
Psychiatrist	○	○	○	○	○
Psychologist	○	○	○	○	○
Neuromonics	○	○	○	○	○
SoundCure	○	○	○	○	○
Acoustic Neuromodulation	○	○	○	○	○
Notched music therapy	○	○	○	○	○
Hearing aid	○	○	○	○	○
Self-administered sound therapy (masking, nature sounds)	○	○	○	○	○
Bio / Neuro feedback, meditation	○	○	○	○	○
Antidepressants	○	○	○	○	○
GABA type drugs	○	○	○	○	○
Retigabine (Trobalt / Potiga)	○	○	○	○	○
Transcranial Stimulation Treatments (rTMS, tDCS, tACS)	○	○	○	○	○
HBOT (Hyperbaric Oxygen Therapy)	○	○	○	○	○
Steroids	○	○	○	○	○
Low-Level Laser Treatment (LLLT)	○	○	○	○	○
Off-label medication	○	○	○	○	○

	Currently undergoing this treatment	Less than 3 months ago	3-6 months ago	6-12 months ago	More than 1 year ago
Surgical procedure	○	○	○	○	○
Acupuncture	○	○	○	○	○
Chiropractor	○	○	○	○	○
Supplements / herbal medicines	○	○	○	○	○
Tinnitus cure eBooks	○	○	○	○	○
Homeopathic treatment	○	○	○	○	○
Other (please specify)	○	○	○	○	○

Your health

*** 22. Did you have any pre-existing health conditions before the onset of tinnitus? (select all that apply)**

- [ ] No existing health conditions
- [ ] Stress
- [ ] Anxiety
- [ ] Depression
- [ ] Panic attacks
- [ ] OCD type conditions
- [ ] Jaw or neck problems
- [ ] Physical disability
- [ ] Alcohol abuse
- [ ] Drug abuse
- [ ] Other (please specify)

Your health (continued)

*** 23. Has tinnitus had an effect on these conditions?**
(phone users - scroll horizontally to see all choices)

	Made a lot worse	Made a little worse	No difference	Made a little better	Made a lot better
No existing health conditions	○	○	○	○	○
Stress	○	○	○	○	○
Anxiety	○	○	○	○	○
Depression	○	○	○	○	○
Panic attacks	○	○	○	○	○
OCD type conditions	○	○	○	○	○
Jaw or neck problems	○	○	○	○	○
Physical disability	○	○	○	○	○
Alcohol abuse	○	○	○	○	○
Drug abuse	○	○	○	○	○
Other (please specify)	○	○	○	○	○

Your health (continued)

*** 24. Has tinnitus caused you any health-related conditions?**

(select all that apply)

- [ ] No health conditions caused
- [ ] Stress
- [ ] Anxiety
- [ ] Panic attacks
- [ ] Depression
- [ ] OCD type condition
- [ ] Insomnia
- [ ] Concentration / focus problems
- [ ] Alcohol abuse
- [ ] Drug abuse
- [ ] Other (please specify)

Your health (continued)

*** 25. Do any of the following dietary items have an effect on your tinnitus?**
(select all that apply)

- [ ] Dietary items do not have an effect
- [ ] Caffeine
- [ ] Sugar
- [ ] Salt
- [ ] Spices / spicy food
- [ ] MSG (Monosodium glutamate)
- [ ] Fatty (junk / takeaway) foods
- [ ] Alcohol
- [ ] Citrus
- [ ] Chocolate
- [ ] Red meat
- [ ] Other (please specify)

Your health (continued)

*** 26. What effect do they have?**
(phone users scroll along to see all choices)

	Make it a lot worse	Make it a little worse	Make it a little better	Make it a lot better
Dietary items do not have an effect	◯	◯	◯	◯
Caffeine	◯	◯	◯	◯
Sugar	◯	◯	◯	◯
Salt	◯	◯	◯	◯
Spices / spicy food	◯	◯	◯	◯
MSG (Monosodium glutamate)	◯	◯	◯	◯
Fatty (junk / takeaway) foods	◯	◯	◯	◯
Alcohol	◯	◯	◯	◯
Citrus	◯	◯	◯	◯
Chocolate	◯	◯	◯	◯
Red meat	◯	◯	◯	◯
Other (please specify)	◯	◯	◯	◯

About your tinnitus
- Last page YEAH!

*** 27. Did you know what tinnitus was before you got it?**

- ◯ Yes
- ◯ Had heard of it but didn't really understand
- ◯ No

*** 28. Where do you perceive your tinnitus?**

- ◯ One ear
- ◯ Both ears
- ◯ More in the brain
- ◯ In the ear/s and the brain
- ◯ Not sure

29. How does your tinnitus behave during the day?

- ◯ It's better in the morning
- ◯ It's better in the evening
- ◯ It fluctuates but there is no pattern
- ◯ It doesn't change at all

*** 30. How often have you heard your tinnitus in the last week?**

(phone users - scroll horizontally to see all choices)

Not heard it	1	2	3	4	5	6	7	8	9	Heard it constantly
◯	◯	◯	◯	◯	◯	◯	◯	◯	◯	◯

*** 31. How often has your tinnitus bothered you over the last week?**

(phone users - scroll horizontally to see all choices)

Not bothered me at all	1	2	3	4	5	6	7	8	9	Bothered me constantly
◯	◯	◯	◯	◯	◯	◯	◯	◯	◯	◯

*** 32. How often has your tinnitus increased feelings of frustration over the last week?**

(phone users - scroll horizontally to see all choices)

No frustration	1	2	3	4	5	6	7	8	9	Constant frustration
◯	◯	◯	◯	◯	◯	◯	◯	◯	◯	◯

33. Please enter your email if you would like to take part in future surveys and make your voice heard amongst the research community

Your details will be kept safe and you can opt out at any time - no spam and we'll ask first before including you on any surveys

Email Address ______________________
