# Supplementary material for: Tinnitus: Characteristics, Need for Therapy, and Therapeutic Outcomes; Results of an International Patient Initiated Platform
Source: Front Neurol. 2022 Jan 20;12:778450. doi: 10.3389/fneur.2021.778450 (PMC8810819; doi:10.3389/fneur.2021.778450)
Supplement: Supplementary file 2 [file Data_Sheet_2.PDF]

TinnitusTalk
SUPPORT FORUM

Investigating Physical Links to Tinnitus

1. About Our Survey

Completion time:
5-10 minutes.

Eligibility criteria:
You must experience tinnitus (hearing a sound when no external sound is present) and have a reasonable understanding of the English language.

Goal:
To better understand how body movements and physical activity can affect tinnitus.

Benefits for tinnitus research:
Your unique tinnitus profile and experiences may reveal clues about the processes involved in tinnitus, and help shape future tinnitus research on the road to a cure.

Privacy:
We value your privacy. Your responses will *not* be matched to you. Your answers may be used for research purposes and in publications in academic journals as well as discussed online and at conferences.

On the final page of this survey, you can optionally fill in your e-mail address so that we can send you the results of the survey once they're published.

Organizer:
The survey is organized by Tinnitus Hub, a UK-based non-profit organization, who is known for operating the Tinnitus Talk Support Forum.

1. Do you agree to the above terms, and do you want to participate in this survey?

- ◯ Yes
- ◯ No

TinnitusTalk
SUPPORT FORUM

Investigating Physical Links to Tinnitus

2. About you

2. How long have you had tinnitus?

	Months	Years
Time		

3. Are you...?

- ◯ Male
- ◯ Female
- ◯ Transgender
- ◯ Other / prefer not to say

4. What is your age?

Move the slider or type in the box.

10 50 100

5. What country do you currently live in?

TinnitusTalk SUPPORT FORUM

Investigating Physical Links to Tinnitus

3. About your tinnitus

6. How loud would you rate your tinnitus?

	Rate the loudness
Over the past week	
On good days	
On bad days	
Right now	

7. What is the cause of your tinnitus?

Please tell us any known causes of your tinnitus, in the order you believe them to be relevant - as many or as few as you are aware of.

	Cause	Diagnose
Primary cause		
Additional cause		
Additional cause		
Additional cause		
Additional cause		

8. Does your tinnitus react to sounds?

- ◯ Some sounds make it worse
- ◯ Some sounds make it worse but some make it better
- ◯ Some sounds can make it better
- ◯ No

9. Do you have any hearing loss?

- ◯ Mild hearing loss
- ◯ Moderate hearing loss
- ◯ Severe hearing loss
- ◯ Not diagnosed but I think I have hearing loss
- ◯ Not diagnosed but I don't think I have hearing loss
- ◯ No

10. How does your tinnitus behave during the day?

- ◯ Generally does not change in volume
- ◯ Generally grows louder as the day progresses
- ◯ Generally gets quieter as the day progresses
- ◯ Changes during the day or over days with no particular pattern

11. Do you get fleeting tinnitus episodes?

Fleeting tinnitus is when your tinnitus loudness increases for a brief moment, your hearing may also become muffled.

- ◯ Daily
- ◯ Weekly
- ◯ Monthly
- ◯ A few times a year
- ◯ Less than a few times a year
- ◯ Never

TinnitusTalk SUPPORT FORUM

Investigating Physical Links to Tinnitus

4. Your physical signs

12. Do you get a stiff neck or have sore neck muscles?

Select all that apply.

- [ ] Yes, after certain physical activity
- [ ] Yes, from bad posture
- [ ] Yes, I have an associated medical condition
- [ ] Yes, from lying in bed / sleeping
- [ ] Yes, my neck movement is restricted due to stiffness
- [ ] No more than I believe is normal

13. Do you get headaches?

- ○ Yes, they feel like they come from the neck
- ○ Yes, they feel like they come from the jaw
- ○ Yes, they come from both the neck and the jaw
- ○ Yes, but I can't pinpoint a cause
- ○ No more than I believe is normal

14. Do you have feelings of fullness in the ears?

Select all that apply.

- [ ] Yes, after activity mainly
- [ ] Yes, after a bad sleep
- [ ] Yes, after listening to some sounds or being exposed to noise
- [ ] Yes, after working at a computer or desk
- [ ] Yes, after periods of stress / anxiety
- [ ] Yes, but I don't know what causes it
- [ ] No

15. Do you grind or clench your teeth?

- ○ Yes, I grind them in my sleep
- ○ Yes, I find that I often clench my teeth without realizing
- ○ Both, I grind in my sleep and clench without realizing
- ○ No, not that I am aware of

16. Does your tinnitus change with any of the below?

Select all that apply.

WARNING - if you perform any of the below your tinnitus may get louder.

- [ ] Pressing the jaw on the side
- [ ] Pushing the jaw backwards
- [ ] Pushing the lower jaw outwards rapidly
- [ ] Pushing your hand against your forehead while resisting with the neck muscles
- [ ] Clenching the teeth together
- [ ] Tilting your head backwards
- [ ] No change with any movements

17. Do you have pain or discomfort in the jaw?

Select all that apply.

- [ ] Yes, my jaw sometimes feels painful
- [ ] Yes, I struggle to fully move my jaw
- [ ] Yes, my jaw sometimes feels tired
- [ ] Yes, muscles around my jaw feel tight or tense
- [ ] Yes, I have several popping and clicking noises in my jaw
- [ ] Yes, I have been diagnosed with TMJ dysfunction
- [ ] No real issues that I am aware of

TinnitusTalk
SUPPORT FORUM

Investigating Physical Links to Tinnitus

5. More about your tinnitus

18. How would you describe your tinnitus?

Select all that apply.

- [ ] A pure tone / single pitch
- [ ] A mixture of tones
- [ ] A low buzzing
- [ ] A high buzzing
- [ ] Electric / interference type sound
- [ ] A low rumbling
- [ ] A static noise
- [ ] Clicking
- [ ] Intermittent beeping (morse code)
- [ ] A whooshing noise - pulsatile
- [ ] A whooshing noise - not in time with the pulse
- [ ] A pulsating sound
- [ ] Other (please specify)

19. How do the following affect your tinnitus?

Stress	
Anxiety	
Waking up from a good nights sleep	
Having a bad nights sleep	
Waking up from napping	
Intense workout (e.g. HiiT, weights, aerobics)	
Moderate exercise (e.g. jogging, cardio)	
Light exercise (e.g. walking, gardening)	

20. Are you able to mask your tinnitus with sounds?

- ◯ Masked by nearly all sounds
- ◯ Masked by things such as TV, music or general background noise
- ◯ Masked by white noise or special masking sounds
- ◯ Masked only by a small selection of sounds
- ◯ Masked only in the shower or by other loud water type sounds
- ◯ Never masked

21. How often are you annoyed by your tinnitus?

	Rate the annoyance
Over the past week	
On good days	
On bad days	
Right now	

22. How aware have you been of your tinnitus over the past week?

Never aware | Aware 50% of the time | Always aware

23. (Optional) If you wish to receive the survey results, provide your e-mail address.

Email Address
